# The Efficacy of Three Modalities of Internet-Based Psychotherapy for Non–Treatment-Seeking Online Problem Gamblers: A Randomized Controlled Trial

**DOI:** 10.2196/jmir.4752

**Published:** 2016-02-15

**Authors:** Amandine Luquiens, Marie-Laure Tanguy, Marthylle Lagadec, Amine Benyamina, Henri-Jean Aubin, Michel Reynaud

**Affiliations:** ^1^ Paul Brousse Hospital, APHP, Villejuif - University Paris-Sud - Inserm U1178 - CESP Department of Addiction Villejuif cedex France; ^2^ URC, Hôpital Pitié –Salpetrière, AP-HP Paris France

**Keywords:** internet-based cognitive behavioral therapy, brief intervention, internet-based randomized controlled trial, problem gambling, non-help seeking, poker, guidance

## Abstract

**Background:**

Internet-based interventions targeted at the most at-risk gamblers could reduce the treatment gap for addictive disorders. Currently, no clinical trial has included non–treatment-seeking patients who have been recruited directly in their gambling environment. This study was the first exclusively Internet-based randomized controlled trial among non–help-seeking problem gamblers with naturalistic recruitment in their gambling environment.

**Objective:**

The aim of this study was to assess the efficacy of three modalities of Internet-based psychotherapies with or without guidance, compared to a control condition, among problem gamblers who play online poker.

**Methods:**

All active poker gamblers on the Winamax website were systematically offered screening. All problem poker gamblers identified with a Problem Gambling Severity Index (PGSI) score of ≥5 were eligible to be included in the trial. Problem gamblers were randomized into four groups: (1) waiting list (control group), (2) personalized normalized feedback on their gambling status by email, (3) an email containing a self-help book to be downloaded with a Cognitive Behavioral Therapy (CBT) program without guidance, and (4) the same CBT program emailed weekly by a trained psychologist with personalized guidance. Efficacy was assessed based on the change in PGSI between baseline and 6 weeks (end of treatment) or 12 weeks (maintenance) and supported by player account-based gambling data automatically collected at the three time points.

**Results:**

All groups met high attrition rates (83%), but the group with guidance had a significantly higher dropout rate than the other three groups, including the control group. Although all groups showed some improvement, with a mean decrease of 1.35 on the PGSI, no significant difference in efficacy between the groups was observed. One-third of the problem gamblers fell below the problem gambling threshold at 6 weeks.

**Conclusions:**

Guidance could have aversively affected problem gamblers who had not sought help. Despite the lack of significant difference in efficacy between groups, this naturalistic trial provides a basis for the development of future Internet-based trials in individuals with gambling disorders. Comorbidities, natural course of illness, and intrinsic motivation seem to be critical issues to consider in future designs.

**Trial Registration:**

ANSM 2013-A00794-41

## Introduction

Despite guidelines for responsible gambling standards [1], online problem and pathological gambling is a growing challenge to health care providers because of its increasing prevalence [2,3] and the limited treatment-seeking by affected subjects. Self-stigma and unawareness of professional sources of help have been described as barriers to accessing the health care system in those with gambling disorders [4]. Online gambling may be more likely to contribute to problem gambling than offline environments [5]. Poker is one of the most popular gambling activities online [2,6]. Poker gamblers seem to be more susceptible to problem gambling compared with the population of all active gamblers [2]. In research to date, larger populations of video poker gamblers have been included in trials aiming to reduce problem gambling, while table poker gamblers have been poorly represented [7,8].

Targeted Internet-based interventions among the most at-risk online gamblers could enhance the efficacy of existing measures and broaden the range of existing sources of help [9]. Therapeutic interventions still have a demonstrated limited effect size in published trials [10]. Most interventions are motivational interventions, cognitive behavioral therapies, or a combination of both [11]. Further data are needed to prioritize one intervention over another and to propose a tailored minimally efficient intervention. In particular, an 8-week Internet-based cognitive behavioral therapy (CBT) program with minimal therapist contact via email and weekly telephone calls of less than 15 minutes has been shown to be effective at reducing pathological gambling, in a population composed of one quarter poker gamblers [8]. Contradictory data are available on the efficacy of motivational support added to CBT strategies [12,13]. However, very short interventions as personalized feedback have been shown to reduce the number of gambling days [14]. Several methodological issues have limited the relevance of behavioral therapy trials among problem gamblers. It may be difficult, for instance, to generalize the findings from patients recruited via advertisements to patients seeking treatment in real-life settings [11].

A fully Internet-based randomized controlled trial is an emerging design that could be particularly pertinent and acceptable in this population, for whom the Internet is the medium of their addictive behavior [15]. Currently, no study has included non-help-seeking patients who have been recruited directly in their gambling environment. Moreover, the Internet-based therapy offers several advantages over face-to-face interventions, including availability, convenience, accessibility, and cost-effectiveness, which are particularly relevant for subjects who are seeking help for their addictive behavior but are not inclined to use traditional services [16]. This study was the first exclusively Internet-based randomized controlled trial among non-help-seeking problem gamblers with naturalistic recruitment in their gambling environment.

The aim of this study was to assess the efficacy of treating online problem gamblers, in particular poker players, with three Internet-based psychotherapy modalities with or without guidance: (1) personalized normalized feedback on their gambling status by email, (2) a self-help book to be downloaded with a CBT program with no guidance, and (3) the same CBT program emailed weekly by a trained psychologist with personalized guidance. Our first hypothesis was that the three Internet-based modalities would be more efficient than the control condition. Our second hypothesis was that less severe problem gamblers would benefit more than more severe ones from modalities requiring less time investment and with no guidance, that is, the personalized normative feedback and the CBT program with no guidance. Our third hypothesis was that more severe gamblers would benefit more from the heaviest intervention requiring more time investment and with guidance.

## Methods

### Participants

All active poker gamblers on the poker gambling service provider, Winamax, were offered screening for problem gambling, that is, a score of ≥5 on the Problem Gambling Severity Index (PGSI). Those identified as problem poker gamblers were proposed to be included in an exclusively Internet-based randomized controlled trial.

### Subject Recruitment

Subjects were considered for enrollment when they started a poker session during the inclusion period from November 13, 2013, to January 16, 2014 (subjects could be included only once). The additional inclusion criteria were age ≥18, completion of registration (ie, an identification card was sent to Winamax to confirm their age), and registration for ≥30 days. On the day after players first opened a poker session during the inclusion period, they were automatically sent an email with a link that they were invited to click and were redirected to an online survey platform hosted by Winamax, where data were collected and then provided to the investigator. Thus, it was a closed survey. The email presented the research team and the perspectives of the study, namely to help identify problem gamblers and offer them an intervention to control their gambling behavior. Before completing the online process, the subjects read a page that contained clear information about the study phase in which they were to be included. The subjects had to read the page to confirm that they agreed and that they understood the study to proceed to the survey. Gamblers were invited to complete the Canadian Problem Gambling Index’s PGSI. If they scored ≥5, they were informed by mail that their scoring could mean they had a gambling problem. They were then invited for inclusion in the randomized control trial with all information on the randomization process and the allocation groups. As a result, the included gamblers were poker gamblers with problem gambling—we use the term “problem poker gamblers” in the paper.

### Interventions

The included gamblers were randomized into four groups following a computer-based randomization process: (1) waiting list (control group), (2) personalized normalized feedback on their gambling status by a preprogrammed email with blanks automatically filled based on the PGSI score, (3) email with a self-help book in a PDF file to be downloaded, containing a CBT program with no guidance, and (4) the same CBT program emailed weekly by a trained psychologist with personalized guidance. The gamblers randomized to the waiting list received an email explaining that they were registered on a waiting list and could contact the research team at the end of the 12-week period if they wanted to benefit from one of the three treatment modalities. The personalized normalized feedback group received an email returning their PGSI score, explaining the corresponding gambling category, and gave prevalence information corresponding to this category, derived from the only available French prevalence data at that time [2]. The therapeutic CBT program was adapted from the Ladouceur self-help book [17], with permission of the author. The program was in accordance with the 6 steps of the self-help program: motivation, financial issues, cognitive distortions, triggers, life reorganization, and relapse prevention. Ladouceur’s 6-step CBT program has shown its efficacy in pathological video poker gambling with media other than a self-help book [18]. Video poker is a casino game played on a computerized console and based on five-card draw poker, which is considered the simplest variant of poker. It is quite different from and reputedly less intimidating than playing table poker with others. More recently, the Ladouceur program was also the basis for the development of another program adapted for Internet use that has shown its efficacy along with additional telephone support, as compared to a waiting list, in a population of pathological gamblers, of which 21.2% were poker gamblers [8]. We chose this program because it has shown its efficacy in poker gamblers, whereas the other self-help book that was assessed in randomized controlled trials at the time of the study, had shown its efficacy in samples largely comprising video lottery gamblers [7,12,13,19]. Owing to the behavioral nature of the interventions, no blinding could be applied. No face-to-face contact or contact by phone was established.

All emails regarding screening, recruitment, intervention, and assessment, except for Group 4 intervention (ie, exchange with the therapist) were automatically generated by the Winamax platform.

### Ethics

Subject consent was obtained as required by local French laws and regulations. The trial has been prospectively registered to the French Medicine Agency, ANSM (ID No. RCB: 2013-A00794-41). The study was authorized by the Comité de Protection des Personnes, as is required for medical intervention research in France. The subjects did not receive any compensation for their participation in this study. Subject anonymity was established and maintained throughout the course of the study, except for Group 4, who agreed to share their email addresses to be able to benefit from the guidance. Before completing the online process, the subjects read a page that contained clear information about the study. The subjects had to read the page to confirm that they agreed and that they had understood the study to proceed to the survey. The subjects received no incentive to respond.

### Sample Size

**Figure 1:**
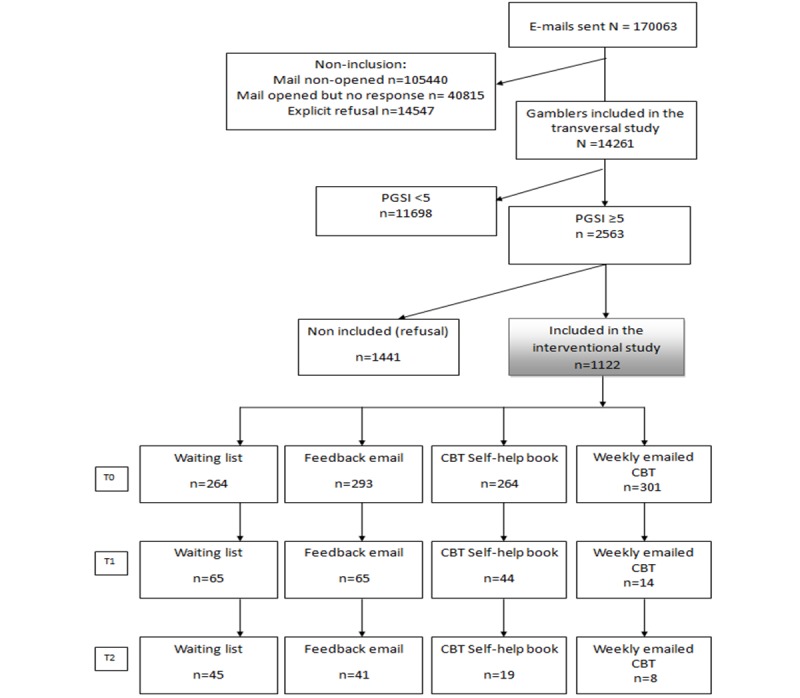
Flow chart.

The sample size for the interventional phase was 992 patients, assuming a standard deviation of PGSI of 8.4, an expected delta of 3 points, and a dropout rate of 50%. We systematically recruited gamblers to be included in the study until we attained the desired sample size for the interventional phase (see Figure 1).

### Settings and Data Collection

After gamblers opened a gambling session on the Winamax website, they were automatically emailed an invitation for inclusion in the study. The assessment of enrolled subjects was completed exclusively online. Player account–based gambling data were prospectively collected automatically at baseline, 6 weeks, and 12 weeks by Winamax and were then retrospectively extracted for the 30-day period before the inclusion day, before week 6 and before week 12. We collected the PGSI at the two endpoints by additional email invitations. Data management and analysis were conducted by the authors. Winamax was contractually commissioned to collect the data, but the authors analyzed the data independently of Winamax.

### Measures

The only additional data collected online involved the PGSI [20]. Since its publication in 2001, the PGSI has become internationally recognized as a robust measure of gambling behavior and has been used in Canada, Australia, Great Britain, Iceland, and Norway. A new screening threshold for this index has recently been proposed [21]. Currie et al recommended eliminating the low-risk and moderate-risk subtypes in favor of two new mid-level categories consisting of low-risk gamblers, defined as a PGSI score of 1-4, and hazardous gamblers, defined as a PGSI score of 5-7. Gamblers with a score of 3 and 4 were a less homogeneous group. Other authors suggest that the PGSI threshold of 8 is too stringent and recommend that 5 and above should define problem gamblers [22]. We chose a priori to use this new threshold of 5 to be more conservative than the previous threshold of 3 for at-risk problem gambling and to avoid inadequate sensitivity of the instrument. In our study, we chose the term “problem gambler” to have an inclusive meaning, designating at-risk and pathological gamblers, as mentioned by the National Center for Responsible gaming [23]. Moreover, the choice of a conservative threshold was driven by a nested clinical trial proposing a therapeutic intervention for the identified problem gamblers. The PGSI is the only self-reporting instrument that has been validated in French to identify problem gamblers. It is short enough to be acceptable and sufficient to screen a large sample of gamblers. We chose this instrument, although the recall period was 12 months, as no other short self-reporting instrument with a shorter recall period was available to identify problem gamblers. There was no randomization of the item order and only a one-page questionnaire. The PGSI is an unusual choice for a primary follow-up variable but is justified by its short length and its use in the only epidemiological French data at this time [2]. It is needed to calculate the sample size and to formulate the personalized normative feedback (Group 2).

Basic sociodemographic and routinely recorded data were extracted from the Winamax player account–based dataset at the three time points. The gambling variables used have previously been reported to be good indicators of problem and pathological gambling (7), and this information is routinely recorded by Winamax. We selected the gambling variables based on ease of their extraction from the Winamax player account–based dataset and according to their reproducibility among other online gambling providers. These criteria were multitabling (playing on multiple tables in the same time) in the past 30 days (yes/no), compulsivity (yes/no) (defined by at least 3 deposits in a period of 12 hours), amount of total deposit in the past 30 days (euros) (an initial deposit is required upon opening the gambling account, which implies that some gamblers could have a null deposit during the study period), mean loss per gambling session including the rake (euros), loss in the past 30 days including the rake (euros), total stakes (euros), number of gambling sessions in the past 30 days, number of gambling days in the past 30 days, and time gambled (hours) in the past 30 days. Two criteria could not be correctly assessed because of technical limits related to a lack of automatic disconnection from the app on some wireless devices, particularly smartphones and tablet computers: time gambled (hours) in the past 30 days and number of gambling days in the past 30 days. We therefore excluded these two criteria from the analysis.

### Statistical Analysis

Included and non-included gamblers were compared using Student’s *t* test for quantitative variables and the chi-square test for qualitative variables. Dropout rates between groups were compared with the chi-square test. The primary outcome was to assess the efficacy of these fully Internet-based interventions based on a comparison of the change in the total PGSI score between baseline and 6 weeks for the Control Group 1 and Groups 2, 3, and 4. The secondary objectives were to compare the changes in the PGSI and other gambling data between baseline and 6 and 12 weeks among the groups. The primary analysis was an intent-to-treat analysis. As stated in the protocol, dropouts were considered failures (no change in total PGSI score). Primary and secondary outcomes were compared to the control group using an analysis of variance with Dunnett’s test for multiple comparisons. To test for a difference in efficacy of the intervention according to the total score at baseline (≥5 and <8 versus >8), an interaction group x total PGSI score at baseline was added to the model. Changes in PGSI and other gambling data within each group were tested with the Wilcoxon signed rank test. The responder rate, defined as the gamblers whose PGSI score decreased by ≥50%, was compared with a chi-square test. To identify potential predictive variables of the response, we performed a multivariate stepwise logistic regression. Variables introduced in the model were demographic and gambling data at inclusion. Finally, as the Ladouceur self-help book emphasizes addressing financial issues, we explored the possibility of a partial efficacy of the interventions on the financial subscore of the PGSI, defined as the sum of the three items of the PGSI regarding financial difficulties (items 1, 4, and 8).

## Results

### Population

Figure 1 shows the CONSORT flowchart of participants. The interventional study was proposed to the 2563 identified problem poker gamblers. Of the identified gamblers, 43.78% (1122/2563) accepted the invitation to participate, were included, and equally randomized into the 4 groups. The baseline characteristics of the included and the non-included problem gamblers are shown in Table 1. The mean age of the included problem gamblers was 34.7 years; the mean age of all of the gamblers (including non-problem gamblers) included in the first cross-sectional phase was similar (35.8 years). Most of them were male, similar to the “all gamblers screened” group (1033/1122, 92.07% and 12,838/14,261, 90.02%, respectively). The mean PGSI score of the included problem gamblers was 9, which is considered pathological gambling. The included problem gamblers were 2 years older and had a more severe gambling addiction and experienced a greater financial impact than the non-included gamblers (significant differences).

**Table 1:** Included and non-included screened problem gamblers characteristics and comparison^a^.

	Included (n=1122)	Non-included (n=1441)	*P* value
Age in years, mean (SD)	34.7 (10.1)	32.55 (9.6)	<.001
Gender, male, n (%)	1033 (92.07%)	1339 (92.92%)	.4
Deposal €, mean (SD)	293.4 (805.7)	212.9 (638.3)	.01
Total loss in €, mean (SD)	180.6 (740.3)	77.3 (652.7)	.0002
Mean loss per gambling session in €, mean (SD)	4.0 (16.7)	2.7 (13.8)	.04
Total stake in €, mean (SD)	1736.1 (5662.6)	1588.1 (11675.8)	.7
Number of gambling sessions, mean (SD)	61.8 (78.6)	58.7 (73.1)	.3
Multitabling, yes, n (%)	898 (80.04%)	1110 (77.03%)	.06
Compulsivity, yes, n (%)	91 (8.11%)	99 (6.87%)	.23
PGSI total score (on 27), mean (SD)	9 (4.7)	7.73 (3.9)	<.001

^a^Student’s *t* test for quantitative variables and chi-square test for qualitative variables.

### Acceptability

Very few gamblers completed the PGSI assessment at the end of the interventions (188/1122, 16.76%; see Table 2). Because the other gambling variables were collected automatically, there were no other missing data. Gamblers who completed the assessment at 6 weeks were older (38.8 vs 33.9 years old), but there were no significant differences in the baseline gambling variables, including the PGSI, except for the mean loss per session, which was lower than in those who did not complete the PGSI at the end of the interventions (-0.11 vs -4.73, *P*=.01). The group who received guidance (Group 4) had the highest dropout rate of the 4 groups, including the control group at 6 and 12 weeks (see Table 2).

**Table 2:** Dropout rate in the randomization groups at 6 and 12 weeks.

Intervention group	Dropout on PGSI at 6 weeks, n (%)	Dropout on PGSI at 12 weeks, n (%)
Waiting list (n=264)	199 (75.4)^a^	219 (83.0)
Feedback email (n=293)	228 (77.8)^a^	252 (86.0)
Self-help CBT book (n=264)	220 (83.3)^a^	245 (92.8) (*P*=.001)
Weekly emailed CBT (n=301)	287 (95.3)^a^	293 (97.3)^a^

^a^ *P* value <.001 (chi-square test).

### Efficacy

The mean PGSI total score decreased significantly between baseline and 6 weeks in the overall sample and within each group, except in the CBT group with weekly emailed guidance (Group 4): mean change -1.35 points (SD 3.8) for the overall sample (see Table 3). Nearly a third of the included problem gamblers assessed at 6 weeks were no longer considered problem gamblers at 6 weeks (PGSI <5). However, we found no significant difference in the changes in total PGSI score at 6 and 12 weeks between the groups.

**Table 3:** PGSI variation score between baseline and 6 weeks by intervention group and by severity.

Intervention group	Mean (SD)		
	All	5≤ PGSI <8 (n=98)	8≤ PGSI (n=91)
Waiting list (n=65)	-1.32 (3.1)	-0.75 (2.4)	-2.03 (3.7)
Feedback email (n=65)	-1.06 (4.1)	-0.74 (2.2)	-1.43 (5.5)
Self-help CBT book (n=44)	-1.73 (4.2)	-0.48 (3.3)	-3.1 (4.7)
Weekly emailed CBT (n=14)	-1.64 (3.9)	-1.25 (3.6)	-1.8 (4.2)

No significant difference was found in the other gambling variables between the groups at 6 and 12 weeks (Tables 4 and 5). Although the findings are statistically insignificant, at 6 and 12 weeks in Groups 1, 3, and 4, total loss and mean loss per session increased, whereas they decreased in the control group (difference between groups not significant). The mean total loss increased by €90 to €99 versus a decrease of €20 in the control group at week 6 and increased by €63 to €737 versus an increase of €3 at week 12. Multitabling decreased significantly within each group at 6 weeks, total deposit in the month decreased significantly within Group 2, and number of gambling sessions decreased significantly within Group 3. At 6 weeks, no significant difference was found in the PGSI score between the groups in the less severe problem gamblers subgroup (5≤ PGSI <8) or the more severe pathological gamblers subgroup (PGSI ≥8) (see Table 3).

**Table 4:** Gambling variables changes at 6 weeks by intervention group (n=1122).

Gambling variables (last 30 days)		Minimum	Median	Maximum	Mean (SD) or %	*P* value within group
**Deposit in €**						
	Waiting list	-5180.00	0.00^b^	1970.00	-9.52 (436.9)	.7
	Feedback email	-3206.00	0.00 ^b^	2487.00	-69.10 (477.4)	.01
	Self-help CBT book	-4774.00	0.00 ^b^	9540.00	33.03 (903.8)	.6
	Weekly emailed CBT	-3645.00	0.00 ^b^	4300.00	-11.99 (474.4)	.70
**Total loss in €** ^a^						
	Waiting list	-5210.95	0.48 ^b^	3348.89	-18.93 (693.7)	.7
	Feedback email	-2377.80	-0.06 ^b^	7518.17	89.64 (953.1)	.11
	Self-help CBT book	-5727.30	3.26 ^b^	13855.52	99.27 (1146.0)	.16
	Weekly emailed CBT	-2614.05	0.00 ^b^	12285.52	93.83 (988.3)	.1
**Mean loss per gambling session** ^a^						
	Waiting list	-206.35	0.01 ^b^	91.12	-1.90 (23.1)	.2
	Feedback email	-69.02	-0.04 ^b^	174.03	1.51 (16.2)	.11
	Self-help CBT book	-162.36	0.02 ^b^	127.93	1.65 (22.3)	.2
	Weekly emailed CBT	-54.54	0.00 ^b^	148.02	1.74 (15.8)	.06
**Total stake in €**						
	Waiting list	-15027.92	-10.88 ^b^	12126.09	-77.70 (2594.7)	.6
	Feedback email	-29009.16	-4.00 ^b^	42677.77	-153.72 (4718.2)	.6
	Self-help CBT book	-39197.70	-30.50 ^b^	129684.50	588.24 (9967.4)	.3
	Weekly emailed CBT	-22094.43	-2.00 ^b^	55041.05	400.32 (4741.9)	.14
**Number of gambling sessions**						
	Waiting list	-448.00	-3.00 ^b^	277.00	-5.18 (70.3)	.23
	Feedback email	-255.00	-2.00	221.00	-4.85 (50.9)	.10
	Self-help CBT book	-452.00	-5.50	403.00	-9.25 (70.9)	.04
	Weekly emailed CBT	-563.00	-3.00	362.00	0.69 (68.3)	.9
**Multitabling, yes**						
	Waiting list	-	-	-	-14%	<.001
	Feedback email	-	-	-	-6%	.03
	Self-help CBT book	-	-	-	-13%	<.001
	Weekly emailed CBT	-	-	-	-9%	.001
**Compulsivity, yes**						
	Waiting list	-	-	-	-0.4%	.83
	Feedback email	-	-	-	-2.4%	.2
	Self-help CBT book	-	-	-	-1.2%	.6
	Weekly emailed CBT	-	-	-	2.3%	.09
**PGSI**						
	Waiting list (n=65)	-13.00	-1.00	6.00	-1.32 (3.1)	<.001
	Feedback email (n=65)	-17.00	-1.00	8.00	-1.06 (4.1)	.04
	Self-help CBT book (n=44)	-17.00	-2.00	8.00	-1.73 (4.2)	.004
	Weekly emailed CBT (n=14)	-11.00	-1.00	6.00	-1.64 (3.9)	.11

^a^A negative value is a worsening, and a positive value is an improvement for the participant.

^b^As the variance is huge on the monetary variables, median is more meaningful than the mean.

The self-help book group had the highest responder rate: 15% (10/65), 17% (11/65), 25% (11/44), and 14% (2/14) in Groups 1, 2, 3, and 4, respectively (no significant difference). Age was the only variable predictive of responder status (*P*=.02); the older gamblers were more likely to decrease their PGSI score by ≥50% at 6 weeks than the younger individuals.

We found no significant difference in the financial subscore of the PGSI between the groups at 6 and 12 weeks.

## Discussion

### Principal Findings

This randomized controlled trial among non–treatment-seeking online problem poker gamblers showed no between-group difference of efficacy of Internet-based interventions compared to placebo. The group with guidance had the highest dropout rate.

### Acceptability

Given the low treatment-seeking status in problem gambling, we ambitiously chose to propose accessing the health care system by proactively inviting problem gamblers screened in their gambling environment to participate in exclusively Internet-based interventions. However, we found limits to the acceptability of these interventions. The dropout rate was very high, although Internet-based randomized trials usually have high dropout rates [15]. Fortunately, there were no missing data on the gambling variables owing to their automatic collection. Engagement information regarding opening of mail and downloading of the CBT book were unfortunately not available and could have provided critical further information on acceptability of the modalities. However, a higher required level of therapeutic personal investment was associated with a higher dropout rate. Similar results have been described previously in problem gamblers, namely, reluctance in completing homework [24]. Another trial among pathological gamblers suggested that “more is not necessarily better,” finding that participants in a brief booster treatment group showed no greater improvement than brief treatment participants without booster [13]. A similar trial, however, that recruited problem gamblers through advertisements showed efficacy of a CBT self-help book enhanced by weekly guidance on the phone by a psychologist [8]. Even if it is traditionally considered that treatments that include guidance seem to lead to better outcomes than unguided treatments [25], our group that received guidance demonstrated a significantly higher dropout rate than the other three groups, including the placebo group. This result could be explained by an aversive effect of guidance among non-help-seeking problem gamblers, possibly because it is too time consuming or too intrusive and is a commitment to someone they have not chosen instead of a commitment to themselves (as in the other groups). Tailored interventions, that is, asking the gambler to choose the level of guidance they could benefit from, could be an innovative way to avoid the aversive effect of guidance.

The proposed interventions could lack an intrinsic motivational component owing to their non-face-to-face nature. Learning during skills-based psychosocial treatments has been shown to be influenced by the intrinsic motivating properties of the treatment context in mental disorders [26]. Intrinsic motivation is specifically and positively associated with more learning, greater persistence of learning, and greater engagement in learning activities. It is known to play a role in treatment success in other psychiatric diseases [26]. A gamification of the modalities could enhance their efficacy. Complete anonymity, or an anonymous feeling for Group 4, in which gamblers shared only their email address, could have lowered the intrinsic motivational component of the proposed program, particularly in non-help-seeking gamblers. Exclusively Internet-based interventions, with no individual contact or only delayed contact by mail (no chatting), could present a poor intrinsic motivational component (eg, no enjoyment or energy of being in a group, no pleasure in social contact, and poor or no experience of therapeutic alliance).

Some trials have proposed financial compensation to lower the dropout rate, through increasing the extrinsic motivation. However, these methods are questionable in patients with a gambling disorder, for whom a monetary gain could interfere with gambling behavior [27], and do not solve the dropout issue [14].

### Accessibility

Khadjesari has already indicated that online trial designs evaluate access to therapeutic material rather than engagement in using it [28]. In regard to increasing accessibility of care, the inclusion rate of almost 50% is high, considering that the problem gamblers screened were not seeking help, and the study showed that proposing a therapeutic intervention to a non-help-seeking problem gambler is realistic. However, the inclusion rate can also be perceived as low if compared to a trial with more traditional recruitment methods. Such care promotion initiatives have been documented to lack efficacy at promoting help-seeking behavior in problem gamblers [29].

**Table 5:** Gambling variables changes at 12 weeks by intervention group (n=1122).

Gambling variables (last 30 days)		Minimum	Median	Maximum	Mean (SD) or n (%)	*P* value within the group
**Deposit in €**						
	Waiting list	-5180.00	0.00	3000.00	-17.67 (573.55)	.03
	Feedback email	-12800.00	-3.00	2150.00	-136.69 (884.45)	<.001
	Self-help CBT book	-4058.00	-10.00	9300.00	-33.41 (801.36)	<.001
	Weekly emailed CBT	-2600.00	-10.00	2710.00	-54.22 (365.61)	<.001
**Total loss in €** ^a^						
	Waiting list	-4990.51	2.31	8333.44	-3.30 (852.54)	.12
	Feedback email	-2315.58	0.68	26580.10	193.78 (1792.40)	.10
	Self-help CBT book	-4051.70	8.00	181410.22	737.06 (11252.29)	.02
	Weekly emailed CBT	-2304.00	2.00	10348.32	63.47 (803.72)	.14
**Mean loss per gambling session** ^a^						
	Waiting list	-1062.72	0.00^b^	176.63	-5.64 (70.02)	.9
	Feedback email	-87.89	0.01	227.21	0.68 (19.09)	.5
	Self-help CBT book	-134.81	0.08	3003.66	11.90 (187.26)	.2
	Weekly emailed CBT	-73.15	0.03	96.21	0.72 (14.10)	.3
**Total stake in €**						
	Waiting list	-20160.14	-63.50	90062.29	143.79 (6345.74)	.007
	Feedback email	-54205.38	-33.21	26282.27	-318.87 (4284.30)	.002
	Self-help CBT book	-62492.55	-36.84	50231.28	-5.36 (7083.45)	.003
	Weekly emailed CBT	-20276.97	-46.50	122346.52	576.16 (8490.70)	<.001
**Number of gambling sessions**						
	Waiting list	-448.00	-7.00	285.00	-11.91 (71.56)	.005
	Feedback email	-262.00	-8.00	318.00	-5.25 (66.27)	<.001
	Self-help CBT book	-381.00	-6.00	356.00	-8.96 (71.42)	.001
	Weekly emailed CBT	-501.00	-8.00	365.00	-6.46 (71.46)	.002
**Multitabling, yes (%)**						
	Waiting list				-18%	<.001
	Feedback email				-13%	<.001
	Self-help CBT book				-18%	<.001
	Weekly emailed CBT				-17%	<.001
**Compulsivity, yes (%)**						
	Waiting list				0.0%	1
	Feedback email				-3.1%	.07
	Self-help CBT book				-0.8%	.7
	Weekly emailed CBT				1.7%	.3
**PGSI**						
	Waiting list (n=45)	-10.00	-3.00	0.00	1.00 (12.00)	.02
	Feedback email (n=41)	-14.00	-3.00	-1.00	0.00 (6.0)	<.001
	Self-help CBT book (n=19)	-9.00	-4.00	-3.00	0.00 (7.0)	.09
	Weekly emailed CBT (n=8)	-8.00	-2.50	-1.00	0.50 (5.0)	.4

^a^A negative value is a worsening, and a positive value is an improvement for the participant.

^b^As the variance is huge on the monetary variables, median is more meaningful than the mean.

### Efficacy

Even if the sample size has been calculated for the primary outcome (PGSI) for which we endorsed a substantial loss to follow-up, the lack of efficacy is supported by the lack of between-groups difference in the secondary criteria, in a very large sample (three time larger per intervention group than the Hodgins’ trial [13]).

There are several explanations for the lack of efficacy of the Internet-based CBT interventions in this trial. First, this trial included non-help-seeking problem gamblers recruited in their gambling environment with no initial involvement in treatment. It has been shown that problem gamblers with higher external motivation for change were less likely to be farther along the stage of change continuum [30]. Low readiness to change could have impacted the efficacy of the interventions. However, in the overall sample, the mean PGSI score at 6 weeks decreased compared with baseline, and one-third of the gamblers were no longer considered problem gamblers at week 6. Another limitation is due to the overlap between the recall periods for the PGSI between the baseline and the two endpoints. This factor could have negatively impacted the sensitivity to change in the assessment. In a future study, an adjusted shortened recall period of the PGSI could prevent that risk of bias. In this particular population of non-help-seeking problem gamblers, a possible therapeutic effect of the inclusion process itself cannot be excluded. The inclusion process required completing the PGSI assessment and receiving an email informing the gambler that their score was above or equal to 5, which defined them as a problem gambler. The email proposed that the individual be included in the study to benefit from therapeutic interventions and thereby recover control of their gambling behavior. The inclusion process was very similar to Group 4, in which gamblers received additional personalized normative feedback. The inclusion process described above is similar to the National Cancer Institute’s smoking cessation counseling recommendations (ie, anticipate, ask, advise, assist, and arrange) proposed to non-treatment-seeking patients consulting a general practitioner for another reason and has proven efficacy [31]. However, this result could also be explained by the natural course of the gambling disorder. High spontaneous remission rates have recently been described in a large Swedish cohort [32]. Gambling disorder seems to benefit from more dynamic change than other addictions, possibly because there is no substance involvement. The efficacy of an intervention could therefore be more difficult to demonstrate. Moreover, if the intervention did not target specifically the poker practice but all kinds of gambling behavior, we could not document gambling behavioral data of other possible gambling practices, except with the PGSI.

In regard to the less severe patients, we chose a conservative threshold of 5 for the PGSI, whereas many trials include patients with a threshold of 3. However, the PGSI is a screening instrument and does not provide a clear diagnosis of gambling disorder. This threshold choice could have biased the results because low-risk or low-problem gamblers have few reasons to change their behavior.

It is also possible that the program is not effective in the gambling population selected in this trial. The program itself could present limits if proposed to any problem gambler; the proposed design is deeply naturalistic, and there were no exclusion criteria except for the legal age limit. Additional psychiatric conditions could have limited the impact of the proposed program, namely, depression and anxiety are frequent comorbidities in problem gamblers and could interfere with work on the cognitive distortions as proposed in our program [33]. For instance, the perceived inability to stop gambling, a frequent gambling-related cognition [34], could be more difficult to challenge in gamblers who experience negative cognition due to depression. However, another Internet-based CBT program has shown efficacy among pathological gamblers, even if depressed [35]. Comorbidities should be considered in a future online trial. Another limitation specific to poker gamblers could be the emphasis on financial issues in the CBT program, because many problem poker gamblers have only moderate financial issues. Manuals other than the Ladouceur program could have been adapted to this study, for instance the Hodgins’ manual, which has shown its efficacy in other populations of pathological gamblers. Our results should not be generalized to other self-help programs. Unfortunately, qualitative material from chat or operator hotlines has not been collected because of technical limitations; participants’ textual feedback could have provided valuable information for better understanding the lack of efficacy of the CBT interventions proposed in this trial [36].

### Conclusion

This first Internet-based randomized controlled trial among non–help-seeking online problem poker gamblers showed a lower acceptability of the modality including guidance compared to the other modalities including placebo. This was possibly due to an aversive effect of guidance in this particular population. We found no significant difference in efficacy between the Internet-based CBT modalities, with or without guidance, compared to the control condition. This naturalistic trial provides a basis for developing future Internet-based trials for individuals with gambling disorders. The natural course of gambling disorders is still poorly documented, and spontaneous changes are a challenge for future assessment of therapeutic interventions. Although Internet-based CBT may enhance access to treatment, it should include intrinsic motivational components to increase engagement in treatment.

## Abbreviations

CBT: cognitive behavioral therapy
PGSI: Problem Gambling Severity Index

## References

[1] National Council on Problem Gambling (2012). Internet responsible gaming standards.

[2] Costes JM (2011). Les niveaux et pratiques des jeux de hasard et d’argent en 2010. Tendances.

[3] Wardle H, Moody A, Spence S, Orford J, Volberg R, Jotangia D, Griffiths M, Hussey D, Dobbie F (2010). National Center for Social Research.

[4] Gainsbury S, Hing N, Suhonen N (2014). Professional help-seeking for gambling problems: awareness, barriers and motivators for treatment. J Gambl Stud.

[5] Griffiths M, Wardle H, Orford J, Sproston K, Erens B (2009). Sociodemographic correlates of internet gambling: findings from the 2007 british gambling prevalence survey. Cyberpsychol Behav.

[6] McCormack A, Shorter GW, Griffiths MD (2013). An examination of participation in online gambling activities and the relationship with problem gambling. J Behav Addict.

[7] Gooding P, Tarrier N (2009). A systematic review and meta-analysis of cognitive-behavioural interventions to reduce problem gambling: hedging our bets?. Behav Res Ther.

[8] Carlbring P, Smit F (2008). Randomized trial of internet-delivered self-help with telephone support for pathological gamblers. J Consult Clin Psychol.

[9] Rodda S, Lubman DI (2014). Characteristics of gamblers using a national online counselling service for problem gambling. J Gambl Stud.

[10] Gainsbury S, Blaszczynski A (2011). A systematic review of Internet-based therapy for the treatment of addictions. Clin Psychol Rev.

[11] Fink A, Parhami I, Rosenthal RJ, Campos MD, Siani A, Fong TW (2012). How transparent is behavioral intervention research on pathological gambling and other gambling-related disorders? A systematic literature review. Addiction.

[12] Hodgins DC, Currie SR, el-Guebaly N (2001). Motivational enhancement and self-help treatments for problem gambling. J Consult Clin Psychol.

[13] Hodgins DC, Currie SR, Currie G, Fick GH (2009). Randomized trial of brief motivational treatments for pathological gamblers: More is not necessarily better. J Consult Clin Psychol.

[14] Cunningham JA, Hodgins DC, Toneatto T, Murphy M (2012). A randomized controlled trial of a personalized feedback intervention for problem gamblers. PLoS One.

[15] Mathieu E, McGeechan K, Barratt A, Herbert R (2013). Internet-based randomized controlled trials: a systematic review. J Am Med Inform Assoc.

[16] Monaghan S, Blaszczynski A (2009). Ontario Problem Gambling Research Centre.

[17] Ladouceur R, Lachance S (2007). Overcoming Your Pathological Gambling: Workbook (Treatments That Work). Overcoming pathological gambling: therapist guide.

[18] Sylvain C, Ladouceur R, Boisvert JM (1997). Cognitive and behavioral treatment of pathological gambling: a controlled study. J Consult Clin Psychol.

[19] Hodgins DC, Currie S, el-Guebaly N, Peden N (2004). Brief motivational treatment for problem gambling: a 24-month follow-up. Psychol Addict Behav.

[20] Ferris J, Wynne H (2001). The Canadian Problem Gambling Index: Final Report, CCSA.

[21] Currie S, Casey DM, Hodgins DC (2010). Canadian Consortium for Gambling Research.

[22] Williams R, Volberg R, Stevens R (2012). Report Prepared for the Ontario Problem Gambling Research Centre & the Ontario Ministry of Health and Long Term Care.

[23] Reilly C, Smith N (2013). The Evolving Definition of Pathological Gambling in the DSM-5.

[24] Dunn K, Delfabbro P, Harvey P (2012). A preliminary, qualitative exploration of the influences associated with drop-out from cognitive-behavioural therapy for problem gambling: an Australian perspective. J Gambl Stud.

[25] Andersson G, Titov N (2014). Advantages and limitations of Internet-based interventions for common mental disorders. World Psychiatry.

[26] Medalia A, Saperstein A (2011). The role of motivation for treatment success. Schizophr Bull.

[27] Brevers D, Bechara A, Cleeremans A, Noël X (2013). Iowa Gambling Task (IGT): twenty years after - gambling disorder and IGT. Front Psychol.

[28] Khadjesari Z, Freemantle N, Linke S, Hunter R, Murray E (2014). Health on the web: randomised controlled trial of online screening and brief alcohol intervention delivered in a workplace setting. PLoS One.

[29] Calderwood K, Wellington WJ (2015). Using Roadside Billboard Posters to Increase Admission Rates to Problem Gambling Services: Reflections on Failure. Health Promot Pract.

[30] Kushnir V, Godinho A, Hodgins DC, Hendershot CS, Cunningham JA (2016). Motivation to quit or reduce gambling: Associations between Self-Determination Theory and the Transtheoretical Model of Change. J Addict Dis.

[31] Schnoll RA, Rukstalis M, Wileyto EP, Shields AE (2006). Smoking cessation treatment by primary care physicians: An update and call for training. Am J Prev Med.

[32] Fröberg F, Rosendahl IK, Abbott M, Romild U, Tengström A, Hallqvist J (2015). The Incidence of Problem Gambling in a Representative Cohort of Swedish Female and Male 16-24 Year-Olds by Socio-demographic Characteristics, in Comparison with 25-44 Year-Olds. J Gambl Stud.

[33] Barrault S, Varescon I (2013). Cognitive distortions, anxiety, and depression among regular and pathological gambling online poker players. Cyberpsychol Behav Soc Netw.

[34] Raylu N, Oei TP (2004). The Gambling Related Cognitions Scale (GRCS): development, confirmatory factor validation and psychometric properties. Addiction.

[35] Carlbring P, Degerman N, Jonsson J, Andersson G (2012). Internet-based treatment of pathological gambling with a three-year follow-up. Cogn Behav Ther.

[36] Griffiths M, Whitty M (2010). Online behavioural tracking in Internet gambling research: Ethical and methodological issues. Int J International Res Ethics.

